# Supplementary material for: Impact of Acute Kidney Injury on Outcomes of Hospitalizations for Heat Stroke in the United States
Source: Diseases. 2020 Jul 15;8(3):28. doi: 10.3390/diseases8030028 (PMC7563434; doi:10.3390/diseases8030028)
Supplement: Supplementary file 1 [file diseases-08-00028-s001.pdf]

Table S1. ICD-9 code for patient characteristics, treatments, and outcomes.

**Characteristics**	
Smoking	305.1, 649.0, 989.84
Alcohol drinking	291.0, 291.1, 291.2, 291.3, 291.4, 291.5, 291.8, 291.81, 291.82, 291.89, 303.00-303.03, 303.90-303.93, 305.00-305.03
Obesity	278.0, 278.00, 278.01, 649.10– 649.14, 793.91, V85.30–V85.4, V85.54
Diabetes Mellitus	249.00–249.31, 250.00–250.33, 648.00–648.04, 249.40–249.91, 250.40–250.93, 775.1
Hypertension	401.1, 401.9, 642.00–642.24, 401.0, 402.00– 405.99, 437.2, 642.10–624.24, 642.70–642.94
Dyslipidemia	272.xx
Hypothyroidism	243.xx, 244.xx
Congestive heart failure	428.xx
Chronic kidney disease	585.1, 585.2, 585.3, 585.3, 585.4, 585.5, 585.6, 585.9
Coronary artery disease	412.xx, 413.xx, 414.xx
Atrial flutter/fibrillation	427.31, 427.32
Complication	
Rhabdomyolysis	728.88
Hyponatremia	276.1
Hypernatremia	276.0
Hypokalemia	276.8
Hyperkalemia	276.7
Hypocalcemia	275.41
Hypercalcemia	275.42
Hypo/hypermagnesemia	275.2
Hypo/hyperphosphatemia	275.3
Metabolic acidosis	276.2
Metabolic alkalosis	276.3
Gastrointestinal bleeding	578.xx
Sepsis	038.0, 038.10, 038.11, 038.19, 038.2, 038.3, 038.4, 038.40, 038.41, 038.42, 038.43, 038.44, 038.49, 038.8, 038.9, 790.7, 117.9, 112.5, 115.04, 115.14, 115.94, 112.81, 112.83, 003.1, 003.21, 036.2, 036.3, 036.0, 036.1, 036.42, 020.2, 022.3, 098.89, 098.84, 098.82, 995.92, 785.52
Ventricular arrhythmia /Cardiac arrest	427.1, 427.41, 427.5
Acute myocardial infarction	410.xx
Treatment	
Invasive mechanical ventilation	96.70-96.73
Blood component transfusion	99.00-99.07
Renal replacement therapy	39.95

**Organ Failure**	**Description**	**ICD-9CM**
Respiratory	Acute respiratory failure	518.81
	Other pulmonary insufficiency, not elsewhere classified. Includes - acute respiratory distress, acute respiratory insufficiency, adult respiratory distress syndrome NEC	518.82
	Acute respiratory distress syndrome after shock or trauma	518.85
	Respiratory distress NOS	786.09
	Respiratory arrest	799.1
	Ventilator management	96.7, 96.70, 96.71, 96.72

Cardiovascular	Shock without mention of trauma	785.5
	Shock unspecified	785.50
	Other shock without trauma (includes hypovolemic Shock)	785.59
	Cardiogenic shock	785.51
	Septic shock	785.52
	Hypotension NOS	458.8, 458.9, 796.3
Renal	Acute kidney injury	584, 584.5, 584.6, 584.7, 584.8, 584.9
Hepatic	Acute hepatic failure or necrosis	570
	Hepatic encephalopathy	572.2
	Hepatitis unspecified	573.3
	Hepatic infarction	573.4
Hematologic	Defibrination syndrome	286.6
	Acquired coagulation factor deficiency	286.7
	Other coagulation defect	286.9
	Thrombocytopenia - secondary or unspecified	287.49, 287.5
Metabolic	Acidosis – metabolic or lactic	276.2
Neurologic	Transient organic psychotic conditions	293, 293.0, 293.1, 293.8, 293.81, 293.82, 293.83, 293.84, 293.89, 293.9
	Anoxic brain injury	348.1
	Acute encephalopathy	348.3, 348.30, 348.31, 348.39
	Coma	780.01
	Altered consciousness - unspecified	780.09
	Electroencephalogram	89.14
	Convulsion	780.39
